# The auto segmentation for cardiac structures using a dual‐input deep learning network based on vision saliency and transformer

**DOI:** 10.1002/acm2.13597

**Published:** 2022-04-01

**Authors:** Jing Wang, Shuyu Wang, Wei Liang, Nan Zhang, Yan Zhang

**Affiliations:** ^1^ Department of Electric Information Engineering Shandong Youth University Of Political Science Jinan China; ^2^ Department of Ecological Environment Statistics Ecological Environment Department of Shandong Jinan China; ^3^ Department of Radiology Beijing Anzhen Hospital Capital Medical University Beijing China; ^4^ Department of Radiology, Shandong Mental Health Center Shandong University Jinan China

**Keywords:** coronary CT angiography, deep learning, self‐attention, transformers, visual attention mechanism

## Abstract

**Purpose:**

Accurate segmentation of cardiac structures on coronary CT angiography (CCTA) images is crucial for the morphological analysis, measurement, and functional evaluation. In this study, we achieve accurate automatic segmentation of cardiac structures on CCTA image by adopting an innovative deep learning method based on visual attention mechanism and transformer network, and its practical application value is discussed.

**Methods:**

We developed a dual‐input deep learning network based on visual saliency and transformer (VST), which consists of self‐attention mechanism for cardiac structures segmentation. Sixty patients’ CCTA subjects were randomly selected as a development set, which were manual marked by an experienced technician. The proposed vision attention and transformer mode was trained on the patients CCTA images, with a manual contour‐derived binary mask used as the learning‐based target. We also used the deep supervision strategy by adding auxiliary losses. The loss function of our model was the sum of the Dice loss and cross‐entropy loss. To quantitatively evaluate the segmentation results, we calculated the Dice similarity coefficient (DSC) and Hausdorff distance (HD). Meanwhile, we compare the volume of automatic segmentation and manual segmentation to analyze whether there is statistical difference.

**Results:**

Fivefold cross‐validation was used to benchmark the segmentation method. The results showed the left ventricular myocardium (LVM, DSC = 0.87), the left ventricular (LV, DSC = 0.94), the left atrial (LA, DSC = 0.90), the right ventricular (RV, DSC = 0.92), the right atrial (RA, DSC = 0.91), and the aortic (AO, DSC = 0.96). The average DSC was 0.92, and HD was 7.2 ± 2.1 mm. In volume comparison, except LVM and LA (*p* < 0.05), there was no significant statistical difference in other structures. Proposed method for structural segmentation fit well with the true profile of the cardiac substructure, and the model prediction results closed to the manual annotation.

**Conclusions:**

The adoption of the dual‐input and transformer architecture based on visual saliency has high sensitivity and specificity to cardiac structures segmentation, which can obviously improve the accuracy of automatic substructure segmentation. This is of gr

## 1 INTRODUCTION

Accurate segmentation of cardiac structures plays an important role in cardiac morphological and functional analysis.^1^, ^2^ Typically, the cardiac structures include the LV, LVM, LA, RV, RA, and AO. For example, LV segmentation can measure end systolic volume (ESV), end diastolic volume, and ejection fraction (EF)^3^; the segmentation of LVM can display the shape and thickness of myocardial wall. These are very important for the evaluation of left ventricular function and the diagnosis of myocardial‐related diseases.^4^, ^5^ In recent years, some studies show that CT can accurately evaluate the shape and function of the right ventricle.^6^, ^7^ However, accurate segmentation of right ventricle is the premise of right heart dysfunction evaluation. More importantly, accurate automatic segmentation and dynamic tracking of cardiac substructures will have a broader prospect for comprehensive evaluation of cardiac morphology and diagnosis of related diseases.^8^

In the past, the method of combining threshold and manual is often used for cardiac substructures segmentation, which is very time‐consuming and highly variable.^9^ There is also research on model‐based CT automatic segmentation algorithm, but only for a single structure, such as LV. With the application of deep learning method, the efficiency of whole heart substructures segmentation is greatly improved, and the accuracy is also improving. Convolutional neural networks (CNNs), in particular fully convolutional networks (FCNs),^10^ such as U‐Net and different variants,^11^, ^12^, ^13^, ^14^ have become dominant in medical image segmentation. Although CNN‐based methods have excellent representation ability, it is difficult to build an explicit long‐distance dependence due to the intrinsic locality of convolution operations.^15^, ^16^, ^17^ Therefore, this limitation of convolution operation raises challenges to learn global semantic information, which is critical for dense prediction tasks like segmentation, especially for target structures that show large inter‐patient variation in terms of texture, shape, and size. To overcome this limitation, various methods have been used for modeling long‐term dependencies. Inspired by the attention mechanism^18^ in natural language processing, existing studies propose alternative architectures, which solely rely on attention mechanisms.^19^, ^20^, ^21^ A typical example is the visual transformer (ViT),^22^ which outperforms ResNet‐based CNN on recognition tasks but at the cost of a large number of the training dataset, which is not always available. Based on this, we attempted to establish self‐attention mechanisms based on CNN features.

In this study, our proposed dual‐input visual saliency and transformer (VST) network has an encoder‐decoder structure. In the encoder, we combine a CNN and a transformer into a hybrid model to make a strong encoder for CCTA image segmentation, specifically, a concise CNN structure is adopted to extract feature maps, and a transformer is used to capture the long‐range dependency. The features with long‐range dependency are fed to the CNN decoder, which performed progressive up sampling to predict the full resolution segmentation map. We segment the cardiac substructures through the model and evaluate the accuracy of the algorithm.

## 2 MATERIALS AND METHODS

### 2.1 Datasets

This retrospective study was approved by the local institutional review board, and informed consent was not required. The information on all images was anonymized before use. In this study, we randomly selected 60 patients’ CCTA images (the best cardiac diastolic period of the R‐R interval) with an average age of 52.6 years (range from 45 to 58 years), including 35 males and 25 females. All experiments follow a fivefold cross‐validation. The size of each image is 512 × 512 pixels and the thickness is 0.75 mm. To ensure the segmentation consistency and less variability, manual segmentation was completed by one person and reviewed by another.

### 2.2 CT acquisition

Using a third‐generation DSCT scanner (SOMATOM Force, Siemens Medical Solutions, Forchheim, Germany), electrocardiography (ECG)‐gated cardiac CT scanning was performed. A retrospective ECG‐gated spiral scan with ECG‐based tube current modulation was applied to multiphase of 0%–90% of the R‐R interval. Automatic exposure control was active, enabling both the adjustment of tube voltage and tube current based on the topogram information. A bolus of 60–70 ml of contrast material (iomeprol; Iomeron 400, Bracco Imaging S.p.A, Milan, Italy) was administered by a power injector (Stellant D, Medrad, Indianola, PA, USA) at 4.5 ml/s followed by 40 ml of saline. An automated bolus tracking system was used to synchronize the arrival of the contrast material with the initiation of the scan. CCTA scan was performed with a tube voltage of 120 kVp, a rotation time of 250 ms, and adaptive tube current (185–380 mA). The effective radiation dose of each scan was calculated by multiplying the dose‐length product by 0.014 mSv/mGy × 1 cm as the constant k‐value. Automatically selected the best cardiac diastolic period, images were reconstructed at a section thickness of 0.75 mm and an increment of 0.6 mm with a Bv40 kernel. The selected FOV was 180mm and the matrix was 512 × 512.

### 2.3 Manual segmentation

**FIGURE 1:**
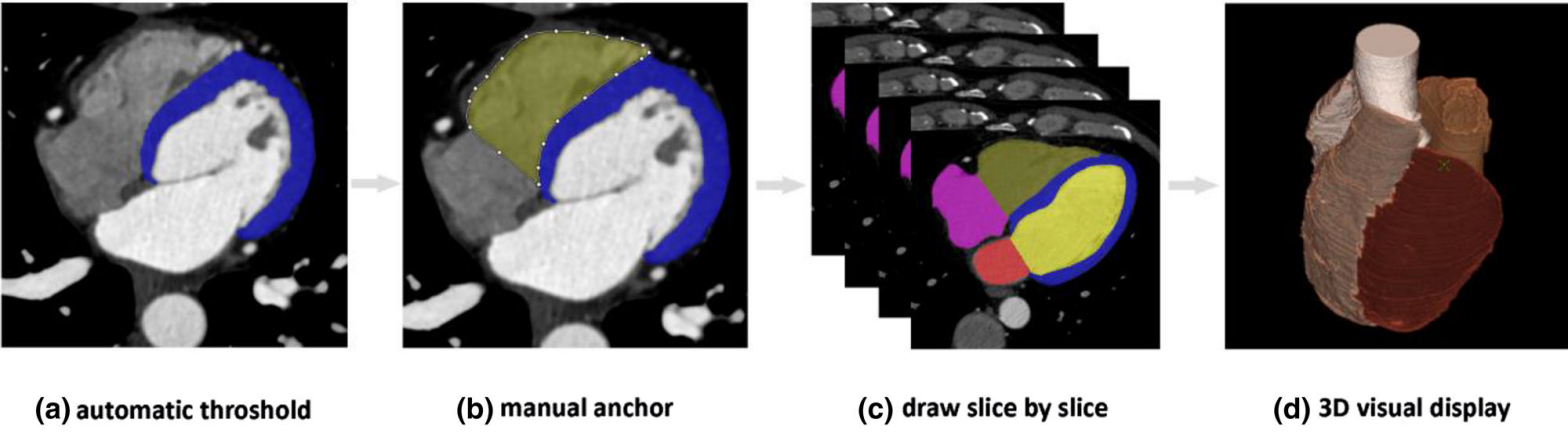
Manual segmentation of cardiac structures. (a) Most of the structures with the large density difference can be segmented through automatic threshold method. (b) In the same slice, the structure with small difference in density from the surrounding can be segmented by manual anchor. (c) Slice by slice to draw until the cardiac structures were marked. (d) The segmentation results can be displayed through 3D visualization to check the accuracy of manual segmentation

In each patient's images, LV, LVM, LA, RV, RA, and AO need to be segmented. The self‐customized labeling tool was used for manual segmentation (Uscube MedLabel, Uscube Science and Technology Co. Ltd., Beijing, China). Most of the structures can be extracted by automatic threshold segmentation. For the structures with inaccurate threshold segmentation, we used the manual anchor method to draw the boundary of tissue structure. We drew slice by slice until all the substructures of the whole heart were marked (as shown in Figure 1). In order to ensure the consistency and less variability of manual image segmentation results, an experienced technician generated the manual segmentation of the data. All the segmentation data were reviewed by a cardiac radiologist.

### 2.4 VST architecture and algorithm

**FIGURE 2:**
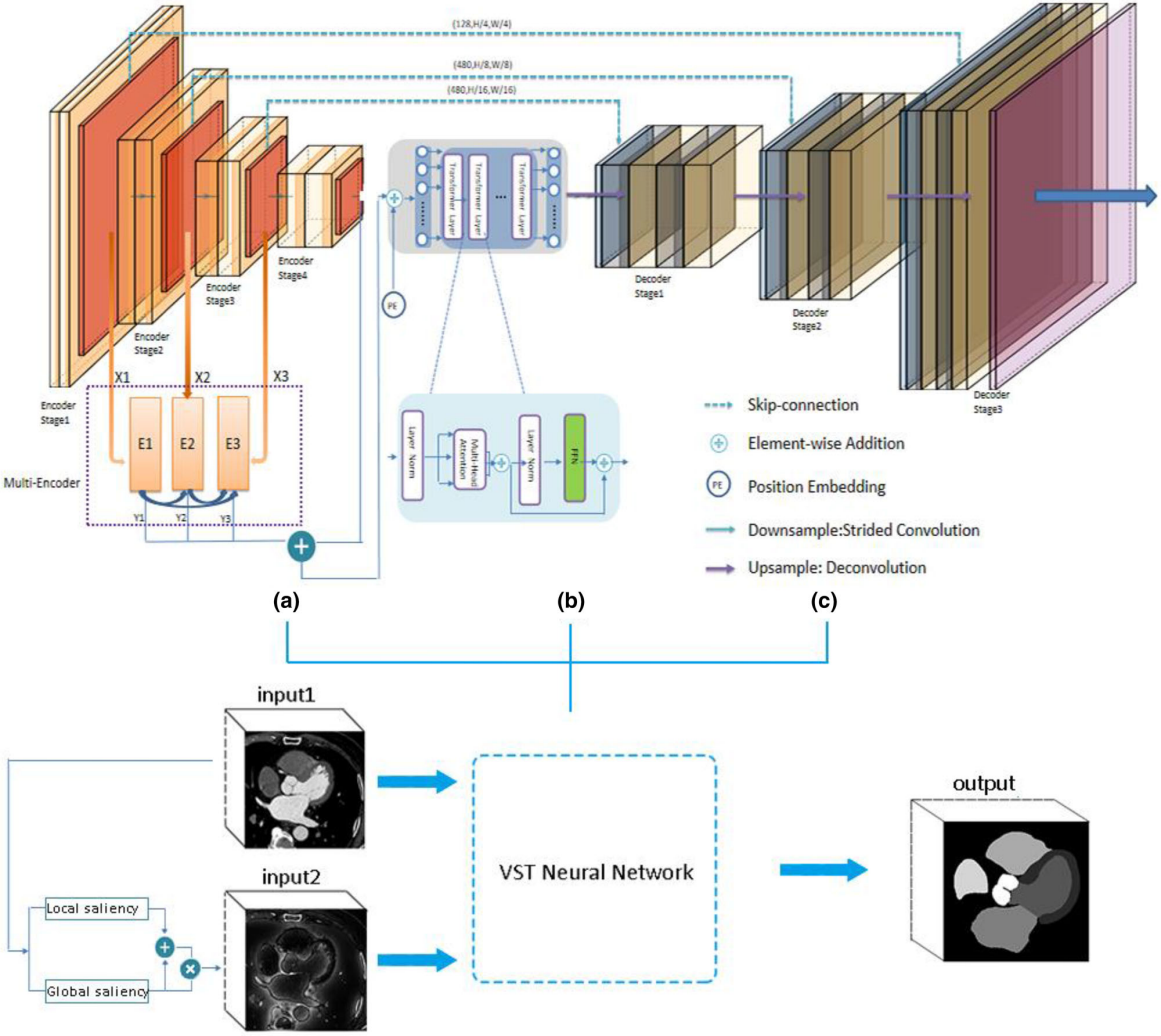
Overall architecture of the proposed visual saliency and transformer (VST). We use input1 and input2 as a dual‐input for tissue contrast: (a) a Convolutional neural networks (CNN) encoder for feature extraction, use a CNN encoder to extract multi‐scale features, and feed the embedded tokens to the transformer; (b) a transformer encoder for long‐range dependency modeling; (c) a CNN decoder for segmentation

An overview of the proposed dual‐input VST was presented in Figure 2. It consisted of a dual‐input: input1 (original CCTA images) and input 2 (obtained from visual attention model) for tissue contrast, a CNN encoder for feature extraction, a transformer encoder for long‐range dependency modeling, and a decoder for segmentation. Next, we described the components of VST in detail.

#### 2.4.1 Dual‐input CCTA images

In order to improve the contrast of the organ tissues, a group of dual‐input CCTA images was employed to provide more feature information to the network. Visual attention model^23^ with cross‐layer saliency optimization was proposed, in which the local saliency and global saliency were extracted. Inspired by this, in our method, the local and global saliency detection was performed based on the contrast of low‐level features respectively; we adopt a multi‐scale transform algorithm to decompose the image into different scales, the local contrast maps are constructed. Based on intensity, texture, and color. First, the input images were decomposed into six spatial scales with the Gaussian pyramid; then three local contrast maps were calculated on each scale to generate eighteen contrast maps; finally, the iterative interpolation algorithm is used to interpolate these contrast maps to form three feature maps ($I_{\mathrm{FM}}^{\prime }$, $T_{\mathrm{FM}}^{\prime }$, $C_{\mathrm{FM}}^{\prime }$), which are combined into the final integrated saliency map (SM), S_Local_

(1) $$S_{\mathrm{Local}}=\sqrt{{\left(N\left(I_{\mathrm{FM}}^{\prime }\right)\right)}^{2}+{\left(N\left(T_{\mathrm{FM}}^{\prime }\right)\right)}^{2}+{\left(N\left(C_{\mathrm{FM}}^{\prime }\right)\right)}^{2}}\;$$

The global saliency is obtained by calculating the differences between the different patches in the image, where the difference is measured by the Euclidean distance between two patches in color space, so the global saliency of pixel *k* is:

(2) $$S_{\mathrm{Global}}\left(k\right)\;=\sum_{j}\mathrm{dis}\left(p_{k},p_{j}\right)\;$$

where $\mathrm{dis}(p_{k},p_{j})$ is the euclidean distance between patches $p_{k}$ and $p_{j}$ in color space.

**FIGURE 3:**
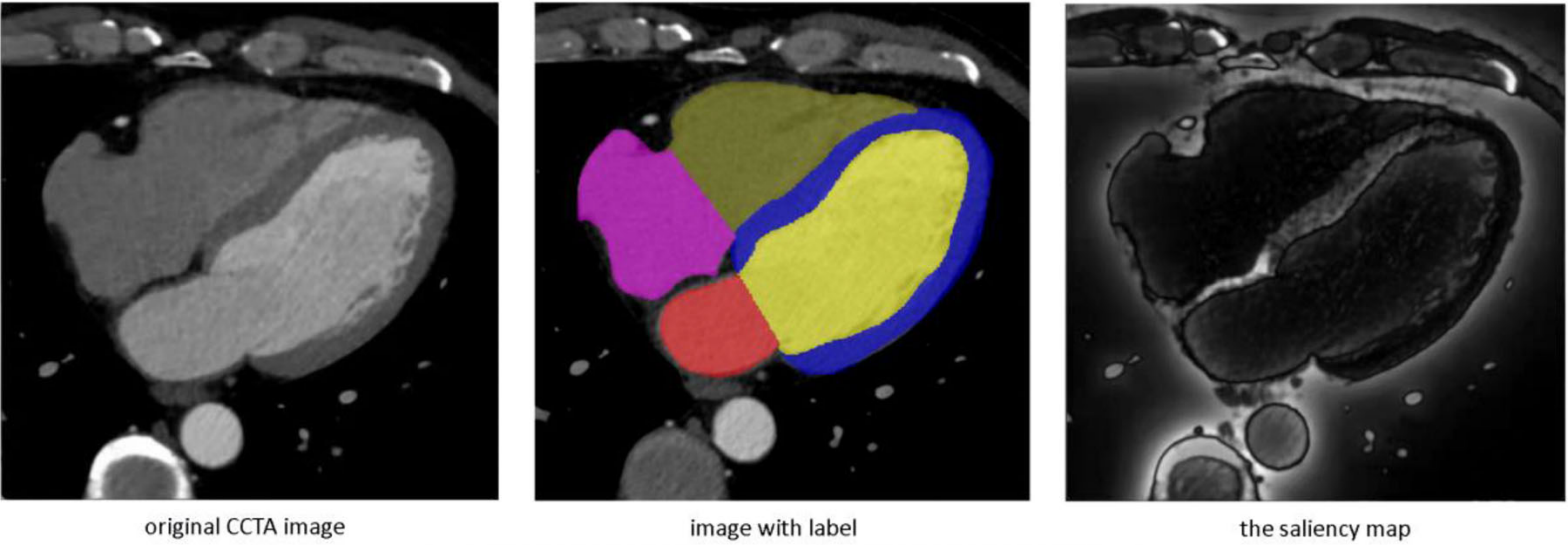
Compare and display the original coronary CT angiography (CCTA) image, the labeled image and the saliency map. From this picture, the addition of the saliency map improved the tissue contrast of the organs in the original CCTA image and also grasped more information about the boundaries. It improved the clarity of the CCTA image boundaries, which played a important role in the following segmentation

Then a weight model was generated based on the obtained local and global SMs, finally the weight model was used as the feedback from local layer to global layer and optimized the global saliency to the final SM. Empirically, the weight values are set θ=0.5. The SM for CCTA image was obtained as following:

(3) $$\omega \;=\;\theta N\left(S_{\mathrm{Local}}\right)+\left(1-\theta \right)N\left(S_{\mathrm{Global}}\right)$$

(4) $$\mathrm{SM}\;=\;\omega \times S_{\mathrm{Global}}$$

where ω is a weighting matrix with the range of value in the matrix was [0, 1]. θ denoted the weight coefficient. *N*(.) denoted normalization. *S*_Global_ and *S*_Local_ denoted the obtained global and local SMs. As shown in Figure 3, SM improved the clarity of the organ boundaries.

#### 2.4.2 CNN‐encoder

Concretely, Given an input image *x* with a spatial resolution of *H* (# of height) × *W*(# of width), depth dimension of *D*(# of slices). Our goal was to predict the corresponding pixel‐wise label map with sizeH×W. The CNN‐encoder consisted of four stages of convolution blocks, which repeated application of two 3 × 3 convolutions (unpadded convolutions), each followed by a Rectified Linear Unit (ReLU) activation and a 2 × 2 max pooling operation with stride 2 for down sampling. In this way, we encoded images into high‐level feature representations, and then the feature maps produced by cnn encoder could be formally expressed as

(5) $$F_{E}^{\mathrm{CNN}}\left(x,\theta \right)\in R^{C\times \frac{H}{2^{s+1}}\times \frac{W}{2^{s+1}}}$$

where *E* indicates the different encoders, θ denotes the parameters of the CNN‐encoder, *S* denotes the stages of the CNN‐encoder, and C denotes the number of channels. At the lowest level, we fused the final output of all encoders in the channel dimension, so that the up‐sampling process could obtain more feature information.

#### 2.4.3 Transformer encoder

The transformer encoder was a composition of an input‐to‐sequence layer and *L* layers of multi‐head self‐attention (MSA) and multi‐layer perceptron (MLP) blocks. Considering that transformer deal with the information in a sequence‐to‐sequence manner, we first flatten the feature maps produced by the CNN‐encoder into a 1D sequence. The CNN encoder designed for CCTA image segmentation down sample a 2D image $X\in R^{H\times W\times 3}$into a feature map $F\in R^{\frac{H}{16}\times \frac{W}{16}\times C}$, we thus decide to set the transformer input sequence length$\frac{H*W}{256}$,This way, the output sequence of the transformer can be simply reshaped to the input feature map of the decoder. To encode the spatial information which was important for CCTA image segmentation, we introduced the learnable position embeddings *X*_pos_, which was added to the feature map $X_{f}$ to form the final sequence input, and the feature embeddings $X_{e}$ can be created as follows:

(6) $$\;X_{e}=X_{f}\;+X_{\mathrm{pos}}$$

MSA was the key components of transformer layers, and MSA was an extension with m independent SA operations (Equations (7) and (8)). Therefore, the output of the *l*th layer could be written as follows:

(7) $$\mathrm{query}=X^{l-1}\;W_{Q},\mathrm{key}=X^{l-1}W_{K},\mathrm{value}=X^{l-1}W_{V}$$

(8) $$\begin{array}{ccc} & & \mathrm{SA}\left(X^{l-1}\right)=X^{l-1}\\ & & \;+\;\mathrm{softmax}\left(\frac{X^{l-1}W_{Q}\left(XW_{K}\right)}{\sqrt{d}}\right)\left(X^{l-1}W_{v}\right) \end{array}$$

(9) $$\mathrm{MSA}\;\left(X^{l-1}\right)=\;\mathrm{Concat}\left(SA_{1},\text{…},SA_{h}\right)W_{O}^{l},$$

where $W_{Q}\setminus W_{k}\setminus W_{V}\in R^{c*d}$ are the learnable parameters of three linear projection layers, and dis the dimension (of query, key, and value). The transformer encoder was composed of *L* transformer layers, which consisted of a multi‐head attention (MHSA) block and a feed forward network. As depicted in Figure 2, the whole calculation could be formulated as:

(10) $$\;X^{l}=\;\mathrm{MSA}\left(X^{l-1}\right)+\mathrm{MLP}\left(\mathrm{MSA}\left(X^{l-1}\right)\right)$$

To thoroughly evaluate the proposed VST framework and validate the performance under different settings, a variety of ablation studies were performed, including: model scaling. (i.e., depth [*L*] and number of heads [*h*]). Two hyper‐parameters, number of heads (*h*), and the number of transformer layers (depth *L*) mainly determine the scale of transformer. We conduct ablation study to verify the impact of transformer scale on the segmentation performance. As shown in Table 1, the network with *h* = 8 and *L* = 8 achieves the best average scores of cardiac structures. Increasing the number of heads (*d*) may not necessarily lead to improved performance (*L* = 8, *h* = 12), and increasing the number of transformer layers (depth *L*) may also not necessarily lead to improved performance (*L* = 12, *h* = 8). Considering that the experiments results and the computation cost, we adopt *L* = 8 and *h* = 8 for all the experiments.

**TABLE 1:** Ablation study on model scaling

		Dice						
Depth (*L*)	Number of heads (*h*)	LVM	LV	LA	RV	RA	AO	Average
6	8	0.83	0.94	0.86	0.89	0.90	0.91	0.89
8	8	0.87	0.95	0.90	0.93	0.91	0.95	0.92
8	12	0.87	0.92	0.90	0.92	0.92	0.92	0.90
12	8	0.84	0.93	0.91	0.88	0.89	0.93	0.89

Abbreviations: AO, aortic; LA, left atrial; LV, left ventricular; LVM, left ventricular myocardium; RA, right atrial; RV, right ventricular.

#### 2.4.4 Decoder

The decoder, a pure CNN architecture, which consisted of stacked up‐sampling steps to decode the hidden feature for outputting. Obviously, we need firstly design a feature mapping module to project the 1D sequence of hidden features back to a standard shape of the lowest CNN feature map and also, the skip connections between encoder and decoder were used to obtain more low‐level details for segmentation task.

### 2.5 Processing and statistical analysis

The VST model was trained from scratch and evaluated using fivefold cross‐validation on the training set. All experiments follow a fivefold cross‐validation, using 80% of images in training and 20% in validation. Specifically, we divided all the obtained slices into five parts, which were not included among each other, four parts were used as the training set, and the remaining one was used as the validation set. Five parts data were taken in turn as the validation set, we calculated the average of the five experimental results. In order to ensure the accuracy of the experimental results, fivefold cross‐validation experiments were repeated five times. We took the average of the five times experimental results as the final results.

In the training stage, we used the Pytorch software packages to train the model, with Adam optimizer (a momentum of 0.1 and an initial learning rate of 0.01). To weigh the balance between training time cost and performance reward, VST was trained for 1000 epochs, and each epoch contains 250 iterations with a batch size of 12. We train our networks with a combination of dice and cross entropy loss:

(11) $$\;L_{\mathrm{total}}=L_{\mathrm{dice}}\;+L_{\mathrm{CE}}$$

To quantitatively evaluate the segmentation results, we calculated the DSC and Hausdorff distance (HD), we also randomly selected a set of test data in fivefold cross‐validation to compare the volume of each structure of the heart between automatic segmentation and manual segmentation and analyzed whether the statistical difference is significant. Statistical analysis was performed using SPSS software (V26.0; SPSS, Chicago, IL, USA). Normal distribution variables were expressed as the mean ± standard deviation (X ± SD), paired sample *t*‐test was used to compare the data of manual segmentation and automatic segmentation. Non‐normal distribution variables were represented by the median and its quartile range (M (Q1, Q3)); the comparison between manual segmentation and automatic segmentation data adopted Wilcoxon signed‐ranks tests with *p* < 0.05 considered significantly different.

## 3 RESULT

Table 2 lists the DSC between reference segmentation and automatic segmentation on CCTA images using five‐fold cross validation to benchmark the segmentation method. Automatic segmentation of all cardiac structures achieved a DSC ≥ 0.87, while the DSC was lowest on the LVM (0.87). If the LVM is excluded, all the structures will achieve a DSC ≥ 0.90. AO had the best performance with DSC = 0.96. In this study, the average DSC was 0.92, and HD was 7.2 ± 2.1 mm. On the whole, this study achieved very good results.

**TABLE 2:** The DSC scores of cardiac structures

Structure	DSC ± SD
Left ventricular myocardium	0.87 ± 0.31
Left ventricular	0.94 ± 0.22
Left atrial	0.90 ± 0.28
Right ventricular	0.92 ± 0.23
Right atrial	0.91 ± 0.34
Aortic	0.96 ± 0.14
Average	0.92 ± 0.25

Abbreviations: DSC, Dice similarity coefficient; SD, standard deviation.

Table 3 shows the volume calculation and statistical analysis of automatic and manual cardiac segmentation in 12 cases. It can be shown from the table that the volume range of each structure of the heart was large among different people. There was no significant statistical difference in cardiac structures except LVM and LA.

**TABLE 3:** Volume statistics results of manual and automatic segmentation of cardiac structures

	Manual segmentation		Auto segmentation		Comparison	
Structure	Range (ml)	M (Q1, Q3)	Range(ml)	M (Q1, Q3)	*Z* value	*p*‐Value
LVM	65.4–128.8	86.1 (74.7, 111.4)	66.2–129.0	86.8 (75.0, 111.8)	–2.084	0.037
LV	45.1–173.5	104.3 (94.2, 152.2)	44.4–172	104.9 (94.2, 154.7)	–0.784	0.433
LA	38.4–98.9	61.4 (45.6, 84.1)	39.0–100.4	61.6 (46.8, 84.8)	–2.041	0.041
RV	62.9–198.9	121.7 (101.4, 171.4)	63.3–200.2	122.4 (103.8, 171.3)	–1.256	0.209
RA	51.1–114.2	67.0 (59.9, 85.5)	52.6–114.6	67.7 (59.3, 87.2)	–1.021	0.307
AO	21.4–48.8	29.9 (26.4, 42.4)	22.1–48.2	29.7 (26.3, 42.8)	–0.315	0.753

Abbreviations: AO, aortic; HD, Hausdorff distance; LA, left atrial; LV, left ventricular; LVM, left ventricular myocardium; RA, right atrial; RV, right ventricular.

## 4 DISCUSSION

Accurate segmentation of cardiac structures plays a more and more important role in cardiac functional assessment. This is not only because the volume change can be measured by whole heart segmentation to calculate ESV, EF, and other indicators.^24^, ^25^ More importantly, the accurate segmentation of the whole heart based on artificial intelligence (AI) on a phase of the R‐R interval plays a vital role and has broad prospects for promoting the development of cardiac functional imaging (such as CT and MR) to track movement, so as to ensure more accurate application in the diagnosis of cardiovascular diseases.^26^, ^27^, ^28^ Because of ECG‐gated and very short acquisition time, CCTA image can well capture the static images of a certain phase in the cardiac cycle and effectively suppress the artifacts of heart beat. On the other hand, intravascular injection of contrast agent can produce obvious contrast between cardiac cavity and myocardium, and there are differences in the concentration of contrast agent between different cardiac cavities. Although some articles have reported the segmentation in dual‐energy non‐contrast enhanced cardiac CT but still based on the contrast‐enhanced CCTA images.^29^ This high‐quality image with contrast difference is more conducive to the segmentation of heart substructures. However, because there is no obvious boundary and contrast difference between the partial substructures of the heart, which brings difficulties to the segmentation automation and accuracy. The application of AI in the field of medical image segmentation provides a very bright direction for cardiac structures segmentation. With the continuous improvement of algorithms and methods, the efficiency and accuracy have been greatly improved.

In this study, we explored using vision saliency‐based transformer architecture for CCTA image segmentation without any pretraining. Multiscale feature representations have shown to benefit various CV tasks.^30^, ^31^, ^32^ Especially, we leverage the multi‐scale feature extracted from CNN to fed to the transformer encoder. VST not only inherits the advantage of CNN for modeling local context information but also leverages transformer on learning global semantic correlations. In our method, we also adopted a vision attention training strategy, which can improve the contrast of the organ tissues. VST achieves superior performances than various competing methods, including CNN‐based self‐attention methods. We apply this method to the segmentation of cardiac structures, and the findings are as following: (1) The lowest Dice coefficient score was LVM (0.87), and the highest was AO (0.96), with an average score of 0.92, showed an overall high level of accuracy. (2) The results of manual segmentation and automatic segmentation were visualized by 3D reconstruction, and they had a high overlap. This meets the clinical requirements and is of great value for clinical application. (3) Most structures volumes of the automatic segmentation and manually obtained reference had small statistical difference and agreed well.

Emerging deep‐learning methods appear as innovative and appealing tools and based on CNN, FCN, U‐net, and various innovative algorithms are applied in this research direction. In this study, we try to improve the segmentation accuracy of our method as much as possible. For quantitative analysis, we use the Dice similarity coefficient to compare our proposed methods with the other state of the art methods. In Table 4, we can see that our method has the higher DSC scores for each of the six cardiac structures, and four scores are the highest among these start‐of‐the‐art methods, the average DSC scores are also the highest among the methods, which also shows the advantages of VST model. In addition, by comparing HD in the table, we can also see our transformer architecture contributes to more accurate segmentation.

**TABLE 4:** Results for each network in Dice similarity coefficient

	DSC							
Network	LVM	LV	LA	RV	RA	AO	average	HD (mm)
Multi‐planar FCNs (2D)^40^	0.85	0.90	0.91	0.88	0.83	0.90	0.88	24.4 ± 11.4
A pipeline of two FCNs (2D)^41^	0.88	0.91	0.92	0.90	0.88	0.93	0.90	25.2 ± 10.8
Multi‐view UNet (2.5D)^42^	0.87	0.93	0.90	0.83	0.88	–	–	31.1 ± 13.2
Faster RCNN and U‐net (3D)^43^	0.82	0.87	0.83	0.90	0.84	0.91	0.86	–
3D FCN (3D)^44^	0.81	0.90	0.79	0.81	0.85	0.72	0.81	29.0 ± 15.8
3D deeply supervised U‐Net (3D)^45^	0.84	0.89	0.89	0.81	0.81	0.87	0.85	44.9 ± 16.1
R‐CNN based on SqueezeNet (3D)^46^	**0.92**	0.85	**0.93**	0.81	0.87	0.91	0.88	–
Ours (VST)	0.87	**0.94**	0.90	**0.92**	**0.91**	**0.96**	**0.92**	7.2 ± 2.1

Abbreviations: AO, aortic; CNN, convolutional neural networks; RCNN, region convolutional neural networks; DSC, Dice similarity coefficient; FCNs, fully convolutional networks; HD, Hausdorff distance; LA, left atrial; LV, left ventricular; LVM, left ventricular myocardium; RA, right atrial; RV, right ventricular. The bold values in the table represent the best performanceof each column.

To illustrate the effectiveness of our approach, performance evaluation of proposed architectures with various normalization techniques are shown in Table 5. U‐Net (using a pure CNN encoder), multi‐U‐Net (using several pure CNN encoders), multi‐U‐Net+transformer (using a hybrid CNN–transformer encoder), multi‐U‐Net+transformer+dual‐input (using a vision saliency‐based hybrid CNN–transformer encoder), in the validation of Multi‐U‐Net, the average DSC performance was improved by 0.01. When compared with U‐Net in segmentation in respect of Cardiac structures. The multi‐U‐Net+transformer showed better performance for average DSC by 0.03, respectively, over Multi‐U‐Net for the segmentation of cardiac structures. The proposed multi‐U‐Net+transformer+dual‐input boosted the performance of segmentation of LVM, LV, LA, RV, RA, AO yielding scores for DSC to 0.87, 0.94, 0.90, 0.92, 0.91, 0.96, respectively. It corroborates that our VST model using a hybrid CNN–transformer encoder has a stronger ability than using a pure CNN encoder to learn effective representations for medical image segmentation.

**TABLE 5:** Performance evaluation of proposed architectures with various normalization techniques

	DSC						
Model	LVM	LV	LA	RV	RA	AO	Average
U‐Net	0.86	0.91	0.82	0.84	0.81	0.84	0.85
Multi‐U‐Net	0.87	0.92	0.74	0.86	0.90	0.85	0.86
Multi‐U‐Net+transformer	0.87	0.92	0.86	0.87	0.89	0.93	0.89
VST (multi‐U‐Net+transformer+dual‐input)	0.87	0.94	0.90	0.92	0.91	0.96	0.92

Abbreviations: AO, aortic; DSC, Dice similarity coefficient; LA, left atrial; LV, left ventricular; LVM, left ventricular myocardium; RA, right atrial; RV, right ventricular; VST, visual saliency and transformer.

In our study, the AO had the highest DSC score, and the LV had the lowest. Several reasons may explain these observations. First, on the CCTA image, the contrast medium concentration in the aorta is very high and uniform, which makes the aorta have a clear boundary with other structures.^33^ The segmentation effect in previous studies was very good. On the premise of good tissue contrast, we added the input of visual significant images, which can better sharpen the structural edge. Therefore, we can get higher DSC score in aortic segmentation. Second, regarding LVM, we elected to include the papillary muscles into the LV label as a common practice,^34^, ^35^ although the papillary muscles include into the LVM label in line with magnetic resonance imaging measurements guidelines.^36^ On the CCTA image, its density is consistent with that of LVM. Due to the high density of LV caused by contrast agent, the papillary muscles contour is often divided into LVM on the visual SM (as shown in Figure 3). From a segmentation standpoint, it likely complicated the automatic delineation of the LVM border, which will adversely affect the result of automatic segmentation. Moreover, the LV has a stronger contrast than the LVM, this effect is much smaller. I think this may explaining the lowest Dice score observed for LVM in the present study. Nevertheless, VST model still achieved a good Dice score compared with other studies, showing its excellent performance in heart substructures segmentation.

**FIGURE 4:**
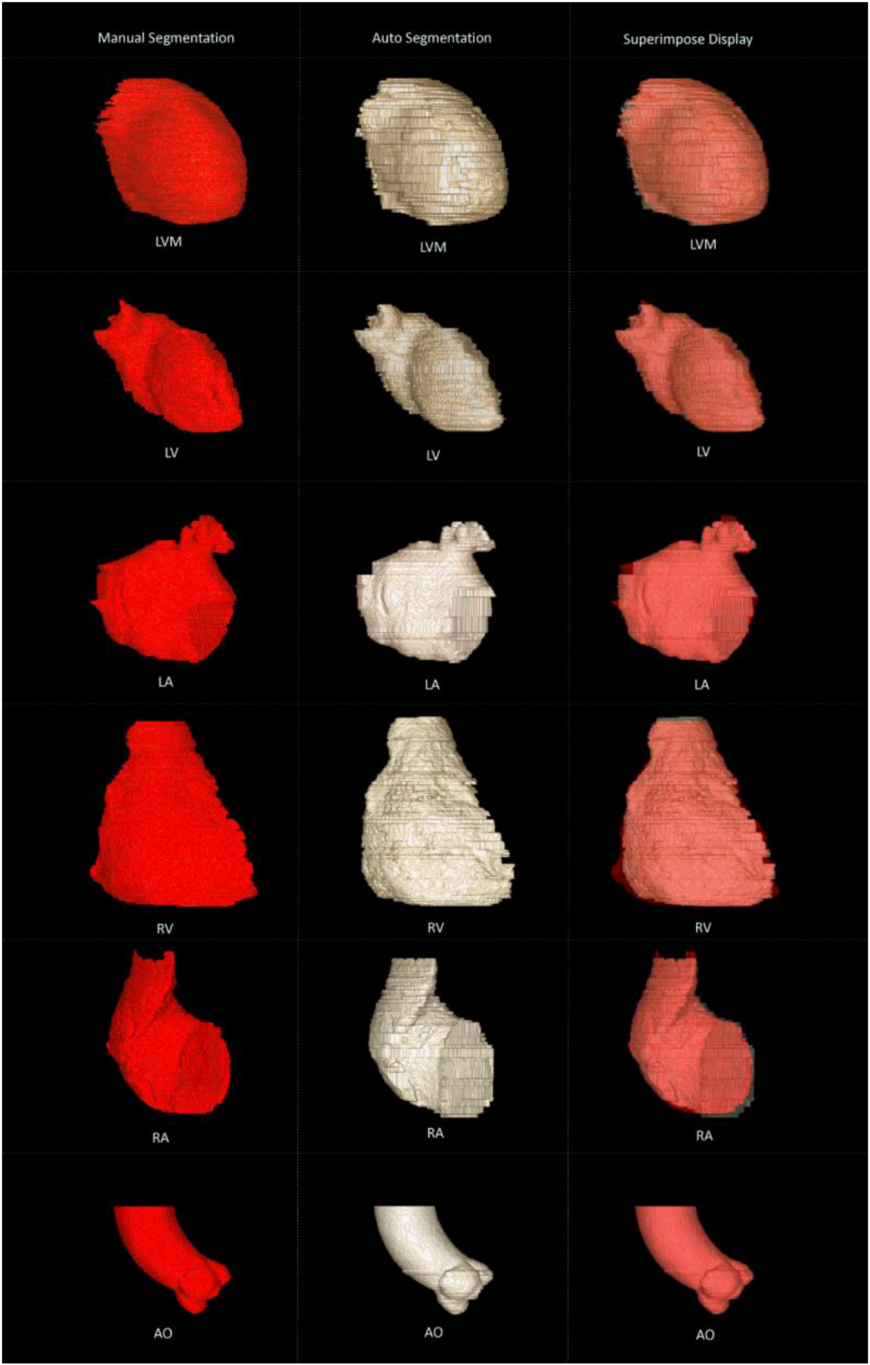
Three dimensional visualization displayed the manually segmented image, visual saliency and transformer (VST) automatically segmented image, and them overlapped. The first column was the manual segmentation image, the second column was the automatic segmentation image, and the third column was the overlay display. In the overlay display image, dark red represented the manual segmentation of non overlapping pixels, while gray green represented the automatic segmentation of non overlapping pixels. On the whole, they had a high overlap

In order to display the effect of segmentation, we performed a three‐dimensional visual display of the results of each heart substructure manually and automatically segmented (as shown in Figure 4). As can be seen from the figure, the automatically segmented image and the manually segmented image can be well overlapped after being superimposed, and only a few edge regions are under or over segmented. In the aspect of visualization, most studies only show the segmentation contour on the 2D image and compare it with the standard value.^37^, ^38^ Morris^39^ only made the overall three‐dimensional visualization of the whole heart structure. In our study, we realized the visual display of the segmented image and the manually segmented image, and overlapped the display, which is very rare in other studies. Three‐dimensional visualization is conducive to more vividly showing the differences of nonoverlapping pixels of the structure, and this result is very necessary for clinical application.

We also calculated the volume of each structure of the heart after automatic segmentation and compared it with manual segmentation results. It shows excellent correlations between manually obtained and deep‐learning predicted volumes for most structures. Although statistically significant absolute differences in volume measurement for the LVM and LA were observed, the mean differences of measurement for all structures were low. This explains the accuracy and practicability of segmentation from a clinical point of view, and the analysis of the volume of different cardiac structures has more practical significance for clinical application. It shows from the study that there is a statistical difference in the volume of LVM, and its DSC is relatively low. This result may have a positive correlation with DSC. About the statistical difference in LA volume, we can find from the image that the LA is connected with superior and inferior vena cava, and heir enhanced density is consistent without obvious boundary. The input visually significant image does not seem to play a role in this boundary enhancement because these structures are continuous, and there is no difference in density. These may be the main factor leading to the difference in volume between automatic segmentation and manual segmentation, or it may be solved by increasing the training data.

In conclusion, we developed a dual‐input deep learning segmentation model based on VST algorithm, which achieved promising results in the segmentation of cardiac structures. This will greatly benefit its potential clinical application. It will surely contribute to establish a more robust and accurate cardiac structure segmentation methods and help to diagnose and treat patients with heart diseases.

## CONFLICT OF INTEREST

The authors declare that there is no conflict of interest that could be perceived as prejudicing the impartiality of the research reported.

## AUTHOR CONTRIBUTIONS

Validation, formal analysis, investigation, writing original draft, writing ‐ review and editing: Jing Wang. Writing ‐ review and editing, software, program debugging: Shuyu Wang. Data collected and statistical analysis: Wei Liang. Formal analysis, writing ‐ review and editing, supervision, resources: Nan Zhang. Software conceptualization, visualization, writing original draft, supervision, and project administration: Yan Zhang.
